# Integration of Steady-State and Temporal Gene Expression Data for the Inference of Gene Regulatory Networks

**DOI:** 10.1371/journal.pone.0072103

**Published:** 2013-08-14

**Authors:** Yi Kan Wang, Daniel G. Hurley, Santiago Schnell, Cristin G. Print, Edmund J. Crampin

**Affiliations:** 1 Auckland Bioengineering Institute, University of Auckland, Auckland, New Zealand; 2 Department of Molecular Medicine and Pathology, University of Auckland, Auckland, New Zealand; 3 Department of Molecular & Integrative Physiology and Department of Computational Medicine & Bioinformatics, University of Michigan Medical School, Ann Arbor, Michigan, United States of America; 4 New Zealand Bioinformatics Institute, Auckland, New Zealand; 5 Maurice Wilkins Centre for Molecular Biodiscovery, Auckland, New Zealand; 6 Department of Engineering Science, University of Auckland, Auckland, New Zealand; 7 Melbourne School of Engineering, The University of Melbourne, Melbourne, Victoria, Australia; 8 National ICT Australia Victoria Research Lab, Canberra, Victoria, Australia; National Institute of Environmental and Health Sciences, United States of America

## Abstract

We develop a new regression algorithm, cMIKANA, for inference of gene regulatory networks from combinations of steady-state and time-series gene expression data. Using simulated gene expression datasets to assess the accuracy of reconstructing gene regulatory networks, we show that steady-state and time-series data sets can successfully be combined to identify gene regulatory interactions using the new algorithm. Inferring gene networks from combined data sets was found to be advantageous when using noisy measurements collected with either lower sampling rates or a limited number of experimental replicates. We illustrate our method by applying it to a microarray gene expression dataset from human umbilical vein endothelial cells (HUVECs) which combines time series data from treatment with growth factor TNF and steady state data from siRNA knockdown treatments. Our results suggest that the combination of steady-state and time-series datasets may provide better prediction of RNA-to-RNA interactions, and may also reveal biological features that cannot be identified from dynamic or steady state information alone. Finally, we consider the experimental design of genomics experiments for gene regulatory network inference and show that network inference can be improved by incorporating steady-state measurements with time-series data.

## Introduction

Determining gene regulatory network structure from gene expression data is one of the most challenging problems in molecular systems biology. Microarray technologies, as well as other newer approaches such as RNA-seq, have been widely used to generate quantitative gene expression data. Typically, experiments measure gene expression following perturbation of target genes (for example following RNAi-mediated gene knock-down or gene deletion), following treatment of cells with a drug or other molecule, or following a change to the cellular environment. Measurements of gene expression are typically conducted at a single time-point, or during successive time-points, after some perturbation. These data are termed steady-state data, and time-series data, respectively. Both types have been used for network inference. Steady-state and time-series data can both provide valuable information about the topology, or ‘wiring diagram’, and dynamics of the gene regulatory network. Compared with steady-state data, time-series data are thought to be more useful for revealing directional interactions to indicate the cause-and-effect relationships among genes [1].

A wide variety of computational algorithms and approaches have been brought to bear on the inference problem from steady-state data, including Bayesian networks [2]–[5], and inference algorithms based on a mutual information (MI) theoretic formalism [6]. Many of these approaches have variants which are adapted for inference from time-series data sets, including dynamic Bayesian network inference [7], [8] and time-dependent MI [9]. For a recent review and discussion of some alternative techniques see [10].

In this work we focus on regression algorithms, in which gene networks are modelled using ordinary differential equations (ODEs) [11]. Some of the earliest work adopting ODEs for temporal expression data was by D'Haeseleer et al. [12]. Many groups have introduced regression algorithms for network inference from time-series data [13]–[18]. ODEs can also be used for regression inference of gene networks from steady-state data. Gardner et al. [19] were amongst the first to demonstrate that steady-state measurements could be used to infer network structure using their network identification by multiple regression (NIR) algorithm. They considered a data set which used plasmids to over-express specific genes in a bacterial model, with measurements taken when the gene expression levels reached new steady state values. Several other groups have also developed similar approaches [20]–[22]. More recently, this approach has been suggested for transcriptomic datasets comprising a set of siRNA knock-down experiments [1]. However, few attempts have been made to infer gene networks for dynamical systems models using steady-state and temporal measurements simultaneously.

In this study we present a regression-based algorithm in which steady-state and time-series datasets can be combined for gene network inference. We base our algorithm on the MIKANA algorithm, which uses a model selection approach for inference of biochemical network models. MIKANA has previously been shown to successfully infer network structures from steady-state and temporal data sets. Comparisons with other gene network inference methods were performed by Hurley et al. [1]. Wildenhain and Crampin [21] used a linear version of the algorithm to reconstruct gene regulatory interactions from simulated steady-state (knock-down) gene expression data. In a separate study, Srividhya et al. [14] used a time-dependent version of the algorithm to identify interactions among reacting components of a biochemical pathway from time-series data. In this work we reformulate these approaches to develop a generalized algorithm for identifying gene regulatory interactions from combinations of both time-series and steady-state gene expression data. We further develop the algorithm to take account of nonlinearities inherent in the regulation of gene expression. We assess the performance of MIKANA for network inference from steady-state data, time-series data and the combined datasets respectively. Performance on different types of data is assessed using synthetic datasets simulated from gene networks under different noise levels and sampling rates. We then apply the algorithm to a human umbilical vein endothelial cell (HUVEC) dataset which combines time-series data following perturbation with the pro-inflammatory growth factor TNF and a steady-state data set comprising response of the cells to knockdown using siRNAs targeting 400 different transcription factors and signalling molecules [1]. Finally we determine whether an efficient experimental design strategy can be determined to improve network inference by combining steady-state and time-series data.

## Results

In this study we compare the performance of three different versions of MIKANA, a regression-based ODE model for gene regulatory network inference. Steady-state MIKANA (ssMIKANA) infers networks from steady-state gene expression data sets. Time-series MIKANA (tsMIKANA) infers networks from temporal gene expression data. A new algorithm, called combined MIKANA (cMIKANA), is developed here to infer gene networks from combined time-series and steady-state data sets. These algorithms and the development of cMIKANA are discussed in the Materials and Methods section.

### Simulation of Microarray gene expression data

To determine whether combining steady-state and time-series data can provide better prediction of gene regulatory interactions, we assessed the performance of network inference with steady-state, time-series and combined datasets by comparing candidate networks inferred from 100-gene simulated datasets against the synthetic networks used to simulate the data. In this work, the *in silico* experiments for simulating gene expression data were designed to mimic the microarray experiments performed previously [1] in generating the steady-state siRNA disruptant (knock-down) data set and TNF perturbation time-series data set for HUVECs analysed below (see Materials and Methods). Steady-state gene expression data were simulated by measuring steady state gene expression levels of other genes while holding the expression level of a target gene at a fixed, reduced level. Time-series data were simulated by sampling the changes in gene expression levels in response to perturbing the initial conditions of the entire network at fixed intervals. This simulates experiments in which multiple genes respond directly to perturbation of the cells, such as action of a compound with multiple targets, or change of environment of the cells. The simulation experiments were repeated several times to generate experimental replicates and the sampling rate for time-series measurements varied, using same network but different initial conditions. Additive noise was added to both steady-state and time-series gene expression data at various levels between 0 and 20% of signal. Full details of the methods used to simulate the gene expression data are described in the Materials and Methods.

### Combining steady-state with temporal gene expression data improves network inference performance from time-series data

**Figure 1:**
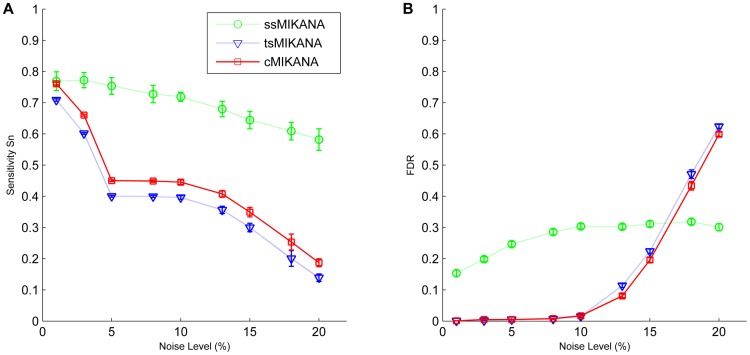
Influence of noise level on the performance of ssMIKANA, tsMIKANA and cMIKANA network inference methods. Different MIKANA network models were inferred from 100-gene scale-free networks. The sensitivity and false discovery rate (FDR) from MIKANA inference methods with steady-state data only (ssMIKANA), time-series data only (tsMIKANA) and the combination of steady-state and time-series data (cMIKANA) are compared. Different noise levels, 1%, 3%, 5%, 8%, 10%, 13%, 15%, 18% and 20%, were added to data, respectively.

In Figure 1 we compare the performance of networks inferred from steady-state, time-series and combined data sets. Inferred networks were scored on an edge-by-edge basis to estimate the sensitivity (Sn; true positive rate) and false discovery rate (FDR). ssMIKANA models were reconstructed from steady-state data only, tsMIKANA models were reconstructed from time-series data only, and cMIKANA models were reconstructed from the combination of the steady-state and time-series data. Each method was assessed using 50 datasets from independent simulations, and in response to varying noise level.

Figure 1 shows that at low noise levels, networks inferred from steady-state data using ssMIKANA have higher sensitivity but also higher FDR than in networks derived using time-series datasets. The performance of ssMIKANA is relatively insensitive to noise compared with tsMIKANA and cMIKANA. This is because ssMIKANA identifies gene interactions from the relative difference between the steady state after perturbation and the initial state (reference level), whereas time-series methods (tsMIKANA and cMIKANA) compare sequential expression levels to calculate the rate of change of expression. As the noise level increases, sensitivity decreases for all of the data types; however, the FDR remains low for time-series data networks.

Although both tsMIKANA and cMIKANA were sensitive to noise, cMIKANA demonstrated better performance compared with tsMIKANA in terms of higher sensitivity while retaining low FDR. We note that at very high noise levels (>20%) FDR increases dramatically for time-series data methods. We have not included any smoothing step in the tsMIKANA or cMIKANA algorithms, and therefore at very high noise levels the differences-based calculation of time derivatives suffers significantly. This may be improved in a straightforward manner by including a data-smoothing step for time-series data.

### A combination of steady-state and time-series gene expression data gives improved network inference over multiple time-series experiments

**Figure 2:**
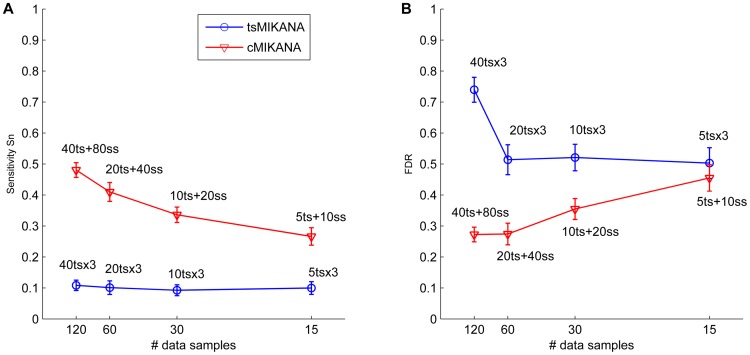
The performance of tsMIKANA and cMIKANA inference methods on the same size of data samples for scale-free networks with 100 genes. The sensitivities and false discovery rates (FDRs) from MIKANA inference methods with time-series data only (tsMIKANA) and with the combination of steady-state and time-series data (cMIKANA) are compared. We reconstructed tsMIKANA network models from time-series datasets – each contained 5, 10, 20 and 40 data samples in 3 replicates, providing 15, 30, 60 and 120 data samples, respectively. We also reconstructed cMIKANA network models from the combined datasets with the same size – containing time-series data sample from 1 temporal experiment (5, 10, 20 and 40 time-series data samples, respectively) and the remainder samples were collected from several knockdown experiments (containing 10, 20, 40 and 80 steady-state data samples, respectively). 10% noise was added to both time-series and steady-state data. Being reconstructed from the same sizes of data, cMIKANA models showed higher sensitivity and relatively lower FDRs compared with tsMIKANA models, suggesting the prediction of gene regulatory interactions could be improved by incorporating steady-state data.

Next we compared networks inferred from multiple time-series data sets with networks generated from a single time-series combined with steady-state data sets, with the same overall number of experimental measurements (120, 60, 30 and 15 samples, respectively). In Figure 2 we compare sensitivity and FDR for inferred network models from data generated with 10% noise from 100-gene networks. At each of four different sampling rates for the temporal data, the combination with steady-state data rather than use of multiple replicates of the time series measurements is shown to give higher sensitivity and lower FDR. We note that tsMIKANA reaches a maximum sensitivity for 4 or more experimental replicates [23], so increasing the number of replicates will not improve the network reconstruction.

Overall, these results show that steady-state and time-series data sets can be combined for network inference and that combining steady-state data and time-series data can raise the sensitivity score for networks identified from time-series data (more true positive edges are identified), while not penalizing the networks by raising the false discovery rate.

### Combining steady-state and time-series data sets does not impair detection of edge directionality

ODE-based network inference approaches assign directional edges from either steady-state data or time-series measurements (unlike most mutual information based approaches for example, which assign non-directional edges irrespective of data type). However, assignment of correct edge direction is thought to be improved using temporal information. We next sought to determine what effect combining steady-state data with temporal data might have on correct assignment of edge direction.

**Figure 3:**
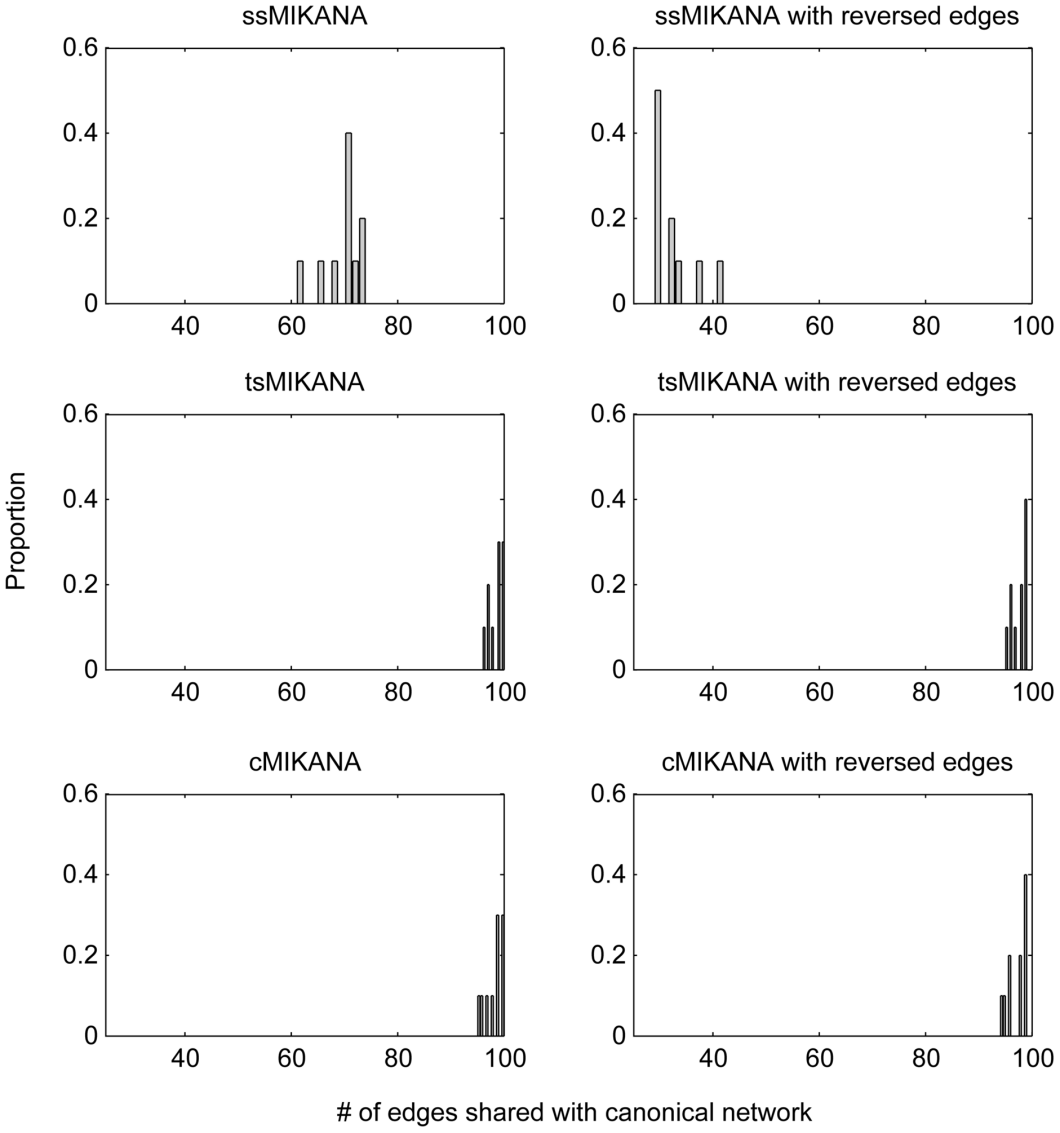
Comparison of the directionality of edges in the ssMIKANA, tsMIKANA and cMIKANA network models. Scale-free networks with 100 genes were generated and related steady-state datasets were simulated. Time-series data were collected at each time point in 10 replicates. 10% noise were added to data. The proportion of edges in the canonical networks found in the forward network models (left column) and in the reversed network models (right column) were computed.

We generated time-series and steady-state data sets from 50 separate simulations for 100-gene networks. Each of these networks had the same connectivity. 10% noise was added to each of data points. We scored the networks inferred using ssMIKANA, tsMIKANA and cMIKANA algorithms for the number of directed edges shared with the networks used to simulate the data. We also scored the number of directed edges shared for the inferred networks in which the direction of each edge was reversed. Figure 3 shows that networks inferred from steady-state data have a lower proportion of edges with correctly identified directionality than for networks identified from time-series data. Comparison with inferred networks with reversed edge direction shows that both data types do however generate a significant number of edges with the wrong directionality.

Finally, the figure shows that by combining steady-state with time series data, networks identified using cMIKANA have approximately the same proportion of correctly identified directed edges. These results confirm that temporal measurements provide directional information for identifying cause-and-effect relationships among genes, but that incorporation of steady-state data does not appear to deteriorate identification of edge directionality.

### Application to time-course and steady-state endothelial datasets

In a study of human umbilical vein endothelial cells (HUVECs), Hurley et al. [1] generated a siRNA disruptant microarray dataset (379 probe sets) from siRNA-mediated knockdowns of 400 specific molecules and transcription factors, and a time-series microarray dataset (234 probe sets), where samples were harvested at 8 time points from a population of HUVECs after being treated with tumour necrosis factor (TNF) (see Methods). To assess the performance of the three versions of MIKANA on real microarray experimental datasets, we reconstructed regulatory networks from these microarray datasets separately and as a combined steady-state and time-series dataset. To carry out a comparison of edges between the various inferred networks, we used the subset of expression data of 50 RNAs that were collected in both the siRNA disruptant and TNF time course datasets (see Table S1). The networks generated using ssMIKANA (130 interactions inferred from the siRNA disruptant data), tsMIKANA (204 interactions inferred from the TNF time course data) and cMIKANA (738 interactions inferred from the combined dataset) for these 50 RNAs were then compared (these networks are presented in Table S2, S3 and S4, respectively).

**Figure 4:**
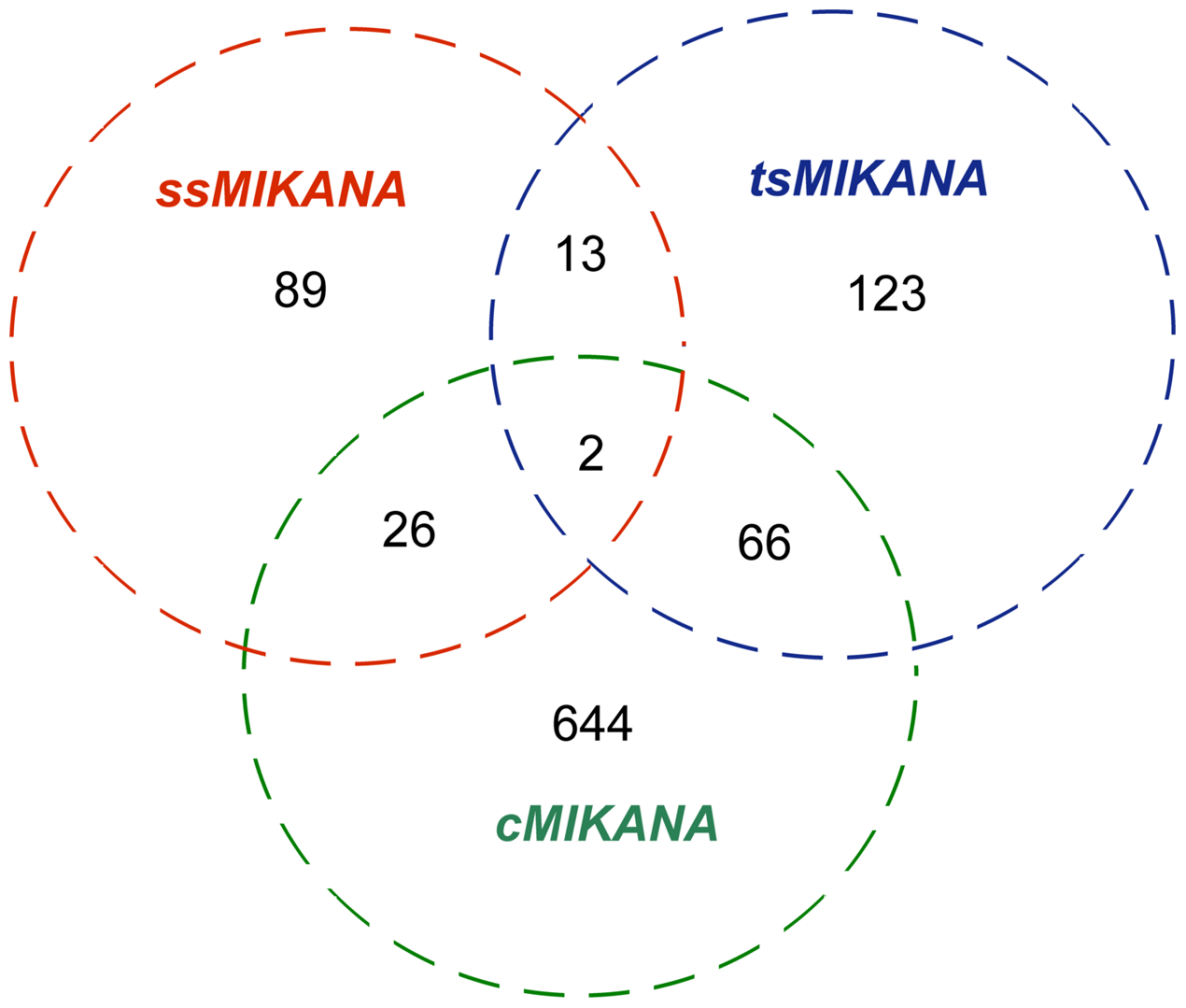
Venn diagram showing the network edges present in three inferred models. The edge-wise comparison between the ssMIKANA, tsMIKANA and cMIKANA models which were reconstructed from the related endothelial dataset is made.

To illustrate the similarities and differences between these three networks, we performed a RNA-to-RNA edge-wise comparison between all three networks. Table 1 summarises the number of directed edges overlapping between each pair of networks, and the Venn diagram shown in Figure 4 illustrates edge overlap in the three different networks. 15 edges were found in both the ssMIKANA (∼12% coverage) and the tsMIKANA (∼7% coverage) networks. Respectively, 28 edges in the ssMIKANA (∼22%) and 68 edges in the tsMIKANA (∼33%) networks could be found in the cMIKANA model, in which only 2 edges were from the overlap of ssMIKANA and tsMIKANA.

**Table 1:** Overlap of edges between all pairs of 50-gene network models.

Overlaps of edges	ssMIKANA	tsMIKANA	cMIKANA	U(TS,SS)
**ssMIKANA**	130	15	28	130
**tsMIKANA**	15	204	68	204
**cMIKANA**	28	68	738	94
**U(TS,SS)**	130	204	94	319

We computed the common edges overlapping among MIKANA network models reconstructed from a siRNA disruptant dataset (ssMIKANA), TNF time course measurements (tsMIKANA) and the combination of the two (cMIKANA). In addition, a union network (U(TS, SS)) was constructed by combining the tsMIKANA and ssMIKANA network models.

To establish whether the network identified using cMIKANA was simply the addition of the ssMIKANA and tsMIKANA network models, we compared the cMIKANA network to the union of the ssMIKANA and tsMIKANA networks. 94 out of 319 interactions (∼30% overlap) in the union network were found to overlap with interactions inferred from the combined data set using cMIKANA.

‘Hub’ genes in regulatory networks are genes with high out-degree, which influence the expression of many other genes. To determine the potential biological significance underlying each of the inferred network models, we ranked genes by out-degree (i.e. the number of target genes in the inferred network) from the three different models. Table 2 summarises the top 10 hub genes (highest out-degree) for each of the three inferred networks. We found 3 hubs (*ID1*, *FOS* and *CFB*) overlapping between ssMIKANA and tsMIKANA network models. These hubs were highly enriched for the regulation of transcription from the Pol II promoter (GO: 0006357 with a Bayes factor of 7) according to the GATHER web tool [24]. Of these, *FOS* was also a hub in the cMIKANA network. Moreover, two other hubs *IL15* (in the top 10 for ssMIKANA) and *HIVEP2* (in the top 10 for tsMIKANA) were also found in the cMIKANA network.

**Table 2:** Top 10 hubs from the ssMIKANA, tsMIKANA and cMIKANA network models respectively, reconstructed from endothelial datasets.

ssMIKANA		tsMIKANA		cMIKANA	
Hubs	# Children	Hubs	# Children	Hubs	# Children
ID1 NM_002165.1_PROBE1	10	DUSP1 NM_004417.2_PROBE1	35	HIVEP2 NM_006734.1_PROBE1	43
CXCL10 NM_001565.1_PROBE1	5	ID1 NM_002165.1_PROBE1	30	F3 NM_001993.2_PROBE1	39
FOS 1227212CB1_PROBE1	5	IRF7 NM_004031.1_PROBE1	30	FOS 1227212CB1_PROBE1	37
NFKB1 NM_003998.1_PROBE1	5	FOS 1227212CB1_PROBE1	20	TLR2 NM_003264.1_PROBE1	37
CD69 NM_001781.1_PROBE1	4	NFATC1 8224569CB1_PROBE1	11	HIVEP2 X65644_PROBE1	33
CFB NM_001710.2_PROBE1	4	IL6 NM_000600.1_PROBE1	10	IL15 2469073CB1_PROBE1	26
CXCL3 NM_002090.1_PROBE1	4	PSMB9 NM_002800.1_PROBE1	9	VCAM1 NM_001078.1_PROBE1	26
ETS1 AK001630_PROBE1	4	BCL3 NM_005178.1_PROBE1	8	RELB NM_006509.1_PROBE1	24
IER3 NM_003897.1_PROBE1	4	HIVEP2 X65644_PROBE1	7	MAP3K8 NM_005204.1_PROBE1	23
IL15 2469073CB1_PROBE1	4	CFB NM_001710.2_PROBE1	6	TUBB2B X79535_PROBE1	23

# Children indicates the number of target genes for each hub gene.

To determine biological function of these genes, we next used GATHER web tool to perform a functional enrichment analysis of the hubs in each network model by comparing the hubs to the Gene Ontology (GO) database. Table 3 summarises the most significant biological annotations (from the GATHER report with Bayes factor >6) of the hubs in the three different networks. Most of the hubs in the three networks (7 hubs in ssMIKANA, 5 hubs in tsMIKANA and 5 hubs in cMIKANA networks) were shown to be highly enriched for immune response (GO: 0006955). This is consistent with the methods used to generate the data sets: through the choice of siRNAs used to generate the steady-state disruptant data set, and through perturbation of the cells using TNF (a cytokine involved in inflammation) for the temporal data (see [1] for details).

**Table 3:** Biological enrichment analysis for the top 10 hubs in the ssMIKANA, tsMIKANA and cMIKANA network models.

ssMIKANA				
Gene Ontology	Genes with Annotation	ln(Bayes factor)	FE: -ln(p value)	FE: -ln(FDR)
GO:0006952: defense response	8	13.02	15.55	11.01
GO:0051244: regulation of cellular physiological process	7	12.56	15.09	11.01
GO:0009607: response to biotic stimulus	8	11.99	14.51	10.84
GO:0006955: immune response	7	10.7	13.22	9.84
GO:0050794: regulation of cellular process	7	10.31	12.83	9.69
GO:0051243: negative regulation of cellular physiological process	5	10.13	12.67	9.69
GO:0050791: regulation of physiological process	10	9.43	11.95	9.12
GO:0050789: regulation of biological process	10	8.34	10.84	8.15
GO:0006954: inflammatory response	4	7.94	10.48	7.97
GO:0043118: negative regulation of physiological process	5	7.92	10.44	7.97
GO:0006366: transcription from Pol II promoter	5	7.79	10.31	7.94
GO:0006916: anti-apoptosis	3	6.82	9.37	7.08
GO:0050896: response to stimulus	8	6.67	9.16	7
GO:0043066: negative regulation of apoptosis	3	6.49	9.03	7
GO:0009611: response to wounding	4	6.48	9	7
GO:0043069: negative regulation of programmed cell death	3	6.46	9	7
TransFac				
V$NFKAPPAB65_01: NF-kappaB (p65)	7	9.66	12.19	6.69
V$NFKB_C: NF-kappaB binding site	5	6.88	9.39	4.59

tsMIKANA				
Gene Ontology	Genes with Annotation	ln(Bayes factor)	FE: -ln(p value)	FE: -ln(FDR)
GO:0009607: response to biotic stimulus	6	7.9	10.25	5.32
GO:0050791: regulation of physiological process	8	6.83	9.17	5.28
GO:0006955: immune response	5	6.4	8.74	5.28
TransFac				
V$NFKB_C: NF-kappaB binding site	5	8.11	10.47	5.02

cMIKANA				
Gene Ontology	Genes with Annotation	ln(Bayes factor)	FE: -ln(p value)	FE: -ln(FDR)
GO:0006955: immune response	5	7.12	9.38	5.1
GO:0006952: defense response	5	6.58	8.83	5.1

The categories in Gene Ontology and TransFac with Bayes factor >6 are summarized and the related number of genes with annotation in the hubs. The Fisher exact p-value (FE: -ln[p value]) and false discovery rate (FE: -ln[FDR]) are presented.

## Discussion

This work has focused on identifying gene regulatory interactions from combinations of steady-state and temporal gene expression data. In real biological regulatory networks, ‘steady-state’ data points can be measured either from perturbation experiments, knock-down ‘disruptant’ data as studied here, or clinical measurements of patients. The measurement of ‘steady-state’ is relative to the experimental time scale and temporal processes that are observed. In reality, however, it is uncertain whether biological data is ever collected at a genuine steady state of the system [13], [25]. For example, although data from siRNA treatment of cells in the laboratory is a type of steady-state data, we recognise that in most cases the siRNA treatments have not persisted long enough for true steady state equilibrium to be reached. In addition to uncertainty about the steady state of some biological data sets, a steady-state experiment does not provide a dynamic description of the system, and is thus arguably less well suited for inference of directed gene networks, which seek to reveal causal regulatory relationships between genes rather than only correlations between gene expression patterns. In this paper we suggest that combining these two types of measurements may improve the prediction of gene regulatory interactions. Unlike other network inference methods, such as Bayesian approaches and mutual information, which require different assumptions and separate formulations for dynamic network inference and steady-state models, analysis of an ODE regression model provides the opportunity to take advantage of both steady-state and temporal data simultaneously. In our study, we implemented a new version of an existing algorithm, which we call cMIKANA, for the reconstruction of networks from combinations of steady-state and time-series data. Our study showed that cMIKANA outperformed the inference from time-series data alone under moderate noise level, limited number of data samples or limited number of experimental replicates.

### Combining steady-state and time-series data sets improves cost-efficiency of microarray experimental design for network inference

The cost and practical complexity of genomic experiments typically limits the number of time-series measurements in a given study to a few time points and a small number of experimental replicates. This constrains the temporal information available for identifying regulatory interactions between genes. We have shown that by combining steady-state data with time-series measurements for network inference it is possible to increase sensitivity without raising the false discovery rate. Furthermore we have shown that addition of this steady-state data does not reduce the ability to correctly determine edge direction in the inferred network. Figure 2 shows for four different overall numbers of measurements that the experimental design in which including steady-state data for the same total number of measurements produces networks with higher sensitivity and lower false discovery rate than is obtained by using the same resources to generate replicate time-series data. This suggests an approach to experimental design for gene network inference in which a combination of time-series and single measurement steady state perturbations are used to generate datasets to optimise network inference. One approach is to generate a single large knock-down data set for a given cell type, that can be used to examine a wide range of different cellular responses by combining with time series data relevant to a specific cellular process.

### Learning biological networks: Application to HUVEC endothelial datasets

Based on the results we achieved from simulated datasets, we tested our new approach using a steady-state siRNA disruptant microarray dataset and a temporal response to perturbation with TNF microarray dataset from HUVECs [1]. Notably we assumed that both datasets interrogated the same biological system, since both experiments focused on extracting information mainly related to inflammatory processes in endothelial cells. However, the two datasets were prepared using different procedures: one was generated to provide as general as possible a network of interaction in endothelial cells that could then be used in the analysis of drug and growth factor response experiments and the other was generated for abstracting interactions related to TNF-regulated RNAs. It is therefore likely that the pathways activated by these interventions, and hence the regulatory networks inferred from these data, described different aspects of the same biological system. To achieve the most relevant comparison, we focused on 50 RNAs that were present and activated in both the siRNA disruptant and TNF time course datasets.

Our analyses of the networks inferred from these two datasets separately and in combination suggest that biologically plausible hub genes were identified in each of the different networks, even though there was relatively low overlap between the hubs and edges identified in the networks determined from the combined data set with those identified from the time-series and steady-state data individually. There are several reasons why the overlap may be low. Using the steady-state formulation of the network inference model, for networks reconstructed using ssMIKANA a gene whose expression profile has high variation across samples and is highly correlated with the expression profile of the ‘regulated’ gene is more likely to be selected as a regulator of that gene in the network model. An edge in this model implies that the variation in the abundance of the regulator RNA can explain (some of) the variance in the abundance of the regulated gene across the experimental samples. Using the temporal formulation of the model, however, for networks inferred using tsMIKANA an edge indicates that the variation in the abundance of a regulating RNA affects the rate of change in abundance of the regulated gene. Another potential reason is that regression methods will select one member of a highly correlated set of genes as a regulator, but different methods select a different member of the same set of highly correlated genes. Greater overlap may therefore be found by preprocessing the data to cluster together gene sets which are highly correlated across all experimental measurements, and to use a single representative from each such highly correlated set for network analysis. Despite these reservations, the inference from the combination of the two datasets using cMIKANA recapitulated 33% of interactions in tsMIKANA and 22% of interactions in ssMIKANA. This suggests that cMIKANA network does not simply represent the union of the tsMIKANA and ssMIKANA networks, but may identify regulatory interactions that were not evident in either the siRNA disruptant network or TNF time course network alone.

In conclusion, we have developed an ODE regression model for reverse-engineering, called cMIKANA, to identify gene regulatory networks from gene perturbation measurements combining steady state and temporal gene expression data. The combined use of time-series and steady-state data outperformed the inference from time-series data only, under moderate noise level. Although different types of genomics experiment measurements may describe different aspects of the regulation underlying the system, our results suggest that combining steady-state and temporal measurements can improve the prediction of gene regulatory interactions and may reveal regulatory information that cannot be observed from either steady-state or time-series data alone. Our results also suggest a potential cost-efficient approach that incorporates steady-state measurements to time-series data sets to improve the design of genomics experiments for gene regulatory network inference.

## Materials and Methods

Gene regulatory networks are usually modeled as a graph of connected nodes, in which nodes represent genes (the expression level of a gene, or the abundance of the related mRNA) and edges represent interactions between genes. We use an ODE formulation as a model for reverse engineering the gene regulatory networks, and as a simulation model to generate synthetic gene expression data sets with which we test our methods. The network simulation, synthetic data generation, network inference and algorithm validation were executed using the computational framework developed by Hurley et al. [1].

### Gene regulatory network inference using MIKANA

MIKANA uses an iterative model selection technique, first proposed by Judd and Mees [26], in order to infer reaction mechanisms for biochemical pathways and networks with little prior information about the underlying pathways. Under different hypotheses about the underlying systems, the ODE-based MIKANA approach was adapted to reconstruct networks from either steady-state or time-series data, and was reformulated to incorporate both time-series and steady-state data. Earlier application of this approach considered models constructed from linear functions of mRNA levels [21]. Here we also introduce a nonlinear basis function to the MIKANA framework, in order to capture the nonlinearity underlying the regulation of gene regulation, as described below. Here we briefly describe the MIKANA algorithm, its formulation for steady-state and time-series data sets, and our new formulation cMIKANA which combines both data types for network inference. General principles describing this approach to network inference can be found in Crampin et al. [27], [28]. Full details of the development of MIKANA and the model selection algorithm that it uses are given in Wildenhain et al. [21], Srividhya et al. [14] and Mourão et al.[29].

The underlying model for MIKANA uses a set of ODEs, one for each gene, describing gene regulation as a function of the level of expression of the other genes in the network, using a linear summation of weighted basis functions:
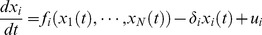
(1.1)

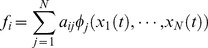
(1.2)where 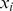 represents the expression level (transcript abundance) of gene 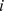. The first term on the right hand side of the equation, 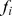 represents the transcription rate of gene 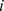, which is determined by the expression level of the genes in the network via function *f_i_* . 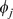 is model design matrix (MDM), which represents the regulatory interaction of parent gene 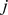 on child gene 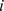. The coefficient 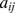 represents the regulatory strength of gene 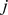 on gene 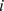. The second term on the right hand side represents the degradation rate of gene 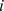; this is assumed proportional to its expression level with proportionality 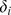. The term 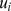 represents the strength of a perturbation applied to the 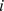
th gene, which moves the system away from its steady state. A model selection approach is used to determine a set of basis functions which best match the data. Using an iterative selection scheme proposed by Judd and Mees [26], the model is established on a gene-by-gene basis by adding the basis function that would make the largest marginal improvement to the model and removing the basis functions that would make the least damage to the approximation. The optimal model size is determined by minimizing a cost function where a compromise between model complexity and a goodness-of-fit is achieved – see [14], [21], [29] for details.

A nonlinear basis function derived from the Hill activation function [30] was adopted in this work to capture the nonlinear regulatory behaviour underlying the data. To simplify computational complexity, we only considered the independent regulatory effect of each individual regulatory gene
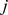
on the target gene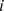, thus the basis function used in this work is
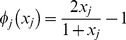
(1.3)


Combining Equations (1.1) and (1.2), the general form of the ODE-based MIKANA can be obtained:
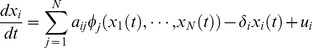
(1.4)


Following Wildenhain and Crampin [21], the steady-state regression form of MIKANA (ssMIKANA) is derived as follows:

Writing 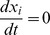 when the system is at steady state and rearranging Equation (1.4) gives:
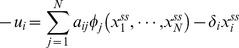
(1.5)where 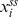 is the expression of gene 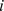 measured at steady state. In real microarray experiments it is often difficult to establish the strength of a perturbation 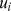 applied to the system. Instead of specifying an arbitrary value for 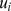, to deal with the situation where, for example, the knockdown perturbation is not known, in ssMIKANA the observation of the perturbed is removed from the regression for that gene.

Following Srividhya et al. [14], we extended the dynamic model (tsMIKANA) for time-series gene expression data to identify dynamic regulatory interactions and to explain the transient response in gene regulatory mechanisms. The tsMIKANA is derived as follows:

Given time-series measurements collected at time points 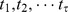, rates of change of expression levels are approximated using 
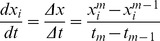
. The general form for the regression then becomes:
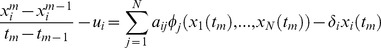
(1.6)


For the time-series microarray experiments considered here, the perturbation is set to zero for tsMIKANA as we assume that the perturbation is to the initial conditions and is not sustained.

Noticing that the right hand sides of Equations (1.5) and (1.6) are identical, we can write the regression model in a form such that steady-state data and time-series measurements can be combined for model fitting simultaneously. Using the nonlinear basis function above, we have the regression for cMIKANA: 
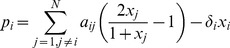
(1.7) where
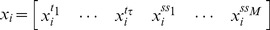


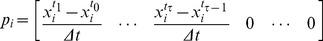



Here 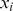 combines the time-series data measured at a series of time points 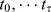 and the steady-state data collected from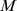 knockdown experiments, for which 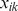 is the expression of the 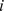
th gene at the 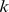
th time point or experiment. 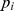 is the combination of the left hand side of Equations (1.5) and (1.6).

### Simulation of gene regulatory networks

We adapted the simulation environment described by Wildenhain and Crampin [21] to generate a gene network, and synthetic gene expression data were then simulated for this network. In the simulation model, the size of the network is defined by the number of genes (nodes) 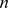. The network topology is defined by the distribution 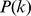 of the number of interactions for each gene, the degree 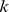, across the nodes in the network. In this work, we have assumed a scale-free topology, where the degree distribution 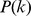 follows a power-law, *P*(*k*)∼*k* ^*−r*^ where 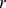 is the degree exponent. Following Barabasi and Albert [31], this topology is constructed following the preferential attachment rule 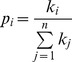, where 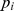 is the probability of adding a new connection to node 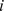, and 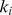 is the degree of node 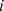. It is assumed that all nodes initially have the same attachment probability, which is randomly generated from a uniform distribution with a range of [0, 1]. Parent and child nodes *a* and *b* are selected at random, and an edge is assigned from *a* to *b* if both 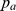 and 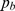 exceed a predetermined threshold. This approach iteratively connects nodes until a specified average degree for 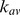 is achieved for the network. We also assign positive and negative regulatory interaction specification to each directed edge in the network, with the ratio of positive to total interactions determined by a predefined parameter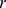. A connection is defined to be positive (activatory regulation) if a random number generated from the uniform distribution [0, 1] is larger than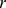. Otherwise the connection is defined to be negative (inhibitory regulation). For the networks simulated in this work, the average connectivity *k_av_*  =  3 and *r*  =  0.65.

### Simulation of gene expression data sets

Given a specified network structure, gene regulatory behaviour can be simulated using the set of ODEs [19] shown in Equation (1.8).
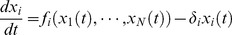
(1.8)


The regulatory effects of positive and negative interactions on gene 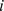
are characterised by 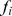 which determines the transcription rate of gene 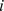 and its degradation rate is 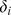. Following the work of Wildenhain and Crampin [21], we used Equation (1.9) to represent the influence of regulator genes acting on gene 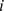


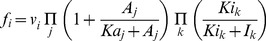
(1.9)where 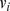 is the basal rate of expression for gene 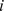, 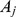 represents the expression level of activator gene 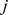 (positive interactions) while 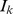 is the expression level of inhibitor gene 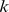 (negative interactions) acting on gene 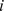 in the network. 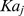 and 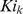 are the saturation constants.

To examine the three variants of the MIKANA network inference approach, two types of numerical experiments were designed for simulating steady-state datasets and time-series datasets of gene expression in response to external stimuli. The numerical experiments were designed in accordance with the microarray studies on HUVECs described in Hurley et al. [1]. Steady-state data were generated to simulate a series of siRNA knockdown experiments in which a different, single gene in the network was perturbed in each experiment. Time-series data were simulated by perturbing the initial conditions of all genes in the network. This was designed to simulate the wide-ranging effect of a broader perturbation, for example triggering inflammatory response.

Two assumptions were made for simulating these experiments: (1) The system is originally at a steady state, which is considered as the reference state for the experiments; (2) the initial concentration of mRNA of a target gene in siRNA knockdown experiments or the mRNA abundances of all genes in a temporal experiment are changed once external stimuli is applied. The simulation has two steps: (i) reference state generation and (ii) microarray experiment simulation. The simulation is executed by MatLab code, which is available from author upon request.

### (i) Reference state generation

Numerical experiments are initiated with all variables 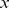 (mRNA concentrations) at reference steady state values. To determine an appropriate reference state, the steady state of the set of ODEs is found by solving with a set of initial conditions being randomly generated from a normal distribution with mean 1000 and standard deviation 1000. The steady-state solution of Equation (1.9) 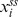 is then used as the reference state 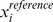 for subsequent experiments.
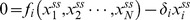
(1.10)


In order to generate numerical data, the parameters in Equations (1.8) and (1.9) were randomly selected from uniform distributions. The range of each distribution was determined empirically in order to achieve a stable model with sufficient variations in simulated data across time: 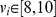, 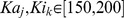, and 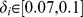.

### (ii) Microarray experiment simulation

#### Simulation of siRNA knock-down

For each knockdown experiment a different gene is perturbed – a target gene is selected and knock-down is simulated by holding the corresponding variable 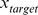 at a fixed, reduced level, such that 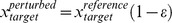. The degree of knock-down 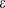 is drawn from a uniform distribution with a range of [0, 1] indicating the percentage by which the expression level of the target gene that would be reduced (by the particular siRNA for example). For the remaining variables, the remaining set of ODEs was integrated.

#### Steady state datasets

To simulate steady state datasets generated from a series of siRNA knock down experiments, the procedure is repeated for each targeted gene in turn, with the remaining N-1 ODEs solved to find the new steady state, generating a set of steady state data.

#### Simulation of broad cellular response to perturbation

To capture the variation in gene expression in response to broader external stimuli, for example cellular response to an inflammatory trigger, we simulated experiments in which all genes in the network were initially perturbed. For each experiment, a set perturbation coefficients 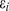 was generated from a uniform distribution with a range of [−1, 1] representing the percentage expression level increase or decrease caused by the perturbation. The ODE system was solved with initial conditions 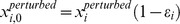 for each gene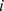.

#### Time-series datasets

To generate time series data a number of gene expression data were collected at sequential time points as the system evolves to a new steady state. The process was repeated 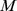 times (replicates) with 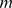 time points, generating an 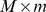 sample time-series dataset.

#### Noise model

To assess the effects of noise on the performance of an ODE-based algorithm we modelled noisy gene expression data 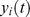 by adding noise that was randomly generated from a Gaussian distribution to the noiseless synthetic data 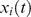:
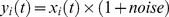
(1.11)where the random variable 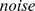 is drawn from Gaussian distribution with a zero mean and a specified standard deviation 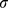 for each noise level.

#### Assessment of network inference

To score the performance of network inference, inferred networks were compared to the synthetic networks used to generate the data by calculating Sensitivity and False Discovery Rate (FDR): 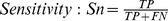
(1.12)

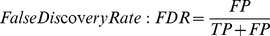
(1.13)where TP stands for true positives, FP is false positives, and FN is false negatives.

### Endothelial cell microarray datasets

In this work, we have used two experimental microarray datasets in HUVECs: siRNA disruptant and TNF time course microarray datasets. These two microarray datasets were prepared previously [1] by using siRNA transfection and TNF treatment, respectively of cultured HUVECs.

To prepare the siRNA disruptant microarray dataset, Hurley et al. had selected 400 siRNA targets, including transcription factors, signalling molecules, receptors and ligands that are related to endothelial cell biology. HUVECs were perturbed by siRNA treatment against each of the selected target RNAs. The global variations in transcript abundance resulting from the siRNA-mediated knockdowns were then measured by the CodeLink UniSet Human 20K Bioarray microarrays. These data are publicly available from the Gene Expression Omnibus (GEO) database with accession number GSE27869. The siRNA disruptant microarray dataset used in this study is a small subset of the data containing 400 samples for 379 RNAs that are particularly selected for their relevance to Rel/NFkB transcription factors, as described in [1].

To prepare the TNF time-series microarray dataset, HUVECs had been treated with the pro-inflammatory growth factor TNF for 24 hours. Samples were then harvested at 0, 1, 1.5, 2, 3, 4, 5 and 6 hours after being treated and were prepared in triplicate. In each of three replicates, the abundance of transcript was measured by CodeLink Uniset microarrays at each time point. These data are publicly available from the GEO database with accession number GSE27870. The TNF-treated time course dataset used in this study is a subset of the data containing 234 differentially expressed RNAs identified by Hurley et al [1] for network analysis. Full experimental details are given in the publication describing the generation of these data sets [1].

## Supporting Information

Table S1 **A list of 50 RNAs that were collected in both the siRNA disruptant and TNF time course datasets.**
(XLSX)

Table S2 **Interactions inferred from the siRNA disruptant data of 50 RNAs using ssMIKANA. 130 interactions were identified by ssMIKANA.** Each interaction is defined by a regulator gene (parent) pointing to a target gene (child) with an interaction coefficient showing the strength of the interaction.
(XLSX)

Table S3 **Interactions inferred from the TNF time course data of 50 RNAs using tsMIKANA.** 204 interactions were identified by tsMIKANA. Each interaction is defined by a regulator gene (parent) pointing to a target gene (child) with an interaction coefficient showing the strength of the interaction.
(XLSX)

Table S4 **Interactions inferred from the combined dataset of 50 RNAs using cMIKANA.** 738 interactions were identified by cMIKANA from the combination of the siRNA disruptant and the TNF time course data of 50 RNAs. Each interaction is defined by a regulator gene (parent) pointing to a target gene (child) with an interaction coefficient showing the strength of the interaction.
(XLSX)

## References

[1] Hurley D, Araki H, Tamada Y, Dunmore B, Sanders D, et al (2012) Gene network inference and visualization tools for biologists: application to new human transcriptome datasets. Nucleic Acids Research 40: 2377–2398. 22121215 10.1093/nar/gkr902 PMC3315333

[2] Heckerman D, Geiger D, Chickering DM (1995) Learning Bayesian networks: The combination of knowledge and statistical data. Machine Learning 20: 197–243.

[3] Zhao Y, Chen MH, Pei B, Rowe D, Shin DG, et al.. (2011) A Bayesian Approach to Pathway Analysis by Integrating Gene-Gene Functional Directions and Microarray Data. Statistics in Biosciences: 1–27. 10.1007/s12561-011-9046-1 PMC3592971 23482678

[4] Berkman O, Intrator N (2007) Robust inference in Bayesian networks with application to gene expression temporal data. Lecture Notes in Computer Science (including subseries Lecture Notes in Artificial Intelligence and Lecture Notes in Bioinformatics). Prague. 479–489.

[5] Friedman N, Linial M, Nachman I, Pe'er D (2000) Using Bayesian Networks to Analyze Expression Data. Journal of Computational Biology 7: 601–620. 11108481 10.1089/106652700750050961

[6] Margolin AA, Nemenman I, Basso K, Wiggins C, Stolovitzky G, et al. (2006) ARACNE: An algorithm for the reconstruction of gene regulatory networks in a mammalian cellular context. BMC Bioinformatics 7.. 10.1186/1471-2105-7-S1-S7 PMC1810318 16723010

[7] Zou M, Conzen SD (2005) A new Dnamic Bayesian Network (DBN) Approach for Identifying Gene Regulatory Networks from Time Course Microarray Data. Bioinformatics 21: 71–79. 15308537 10.1093/bioinformatics/bth463

[8] Dojer N, Gambin A, Mizera A, Wilczynski B, Tiuryn J (2006) Applying dynamic Bayesian networks to perturbed gene expression data. BMC Bioinformatics 7: 249. 16681847 10.1186/1471-2105-7-249 PMC1513402

[9] Zoppoli P, Morganella S, Ceccarelli M (2010) TimeDelay-ARACNE: Reverse engineering of gene networks from time-course data by an information theoretic approach. BMC Bioinformatics 11: 154. 20338053 10.1186/1471-2105-11-154 PMC2862045

[10] Penfold CA, Wild DL (2011) How to infer gene networks from expression profiles, revisited. Interface Focus 1: 857–870. 23226586 10.1098/rsfs.2011.0053 PMC3262295

[11] De Jong H (2002) Modeling and simulation of genetic regulatory systems: A literature review. Journal of Computational Biology 9: 67–103. 11911796 10.1089/10665270252833208

[12] D'Haeseleer P, Wen X, Fuhrman S, Somogyi R. Linear modeling of mRNA expression levels during CNS development and injury; 1999; University of New Mexico, Department of Computer Science, Albuquerque 87131, USA. 41–52. 10.1142/9789814447300_0005 10380184

[13] Bonneau R, Reiss DJ, Shannon P, Facciotti M, Hood L, et al (2006) The inferelator: An algorithn for learning parsimonious regulatory networks from systems-biology data sets de novo. Genome Biology 7: R36. 16686963 10.1186/gb-2006-7-5-r36 PMC1779511

[14] Srividhya J, Crampin EJ, McSharry PE, Schnell S (2007) Reconstructing biochemical pathways from time course data. Proteomics 7: 828–838. 17370261 10.1002/pmic.200600428

[15] Tegnér J, Yeung MKS, Hasty J, Collins JJ (2003) Reverse engineering gene networks: Integrating genetic perturbations with dynamical modeling. Proceedings of the National Academy of Sciences 100: 5944–5949. 10.1073/pnas.0933416100 PMC156306 12730377

[16] van Someren EP, Vaes BLT, Steegenga WT, Sijbers AM, Dechering KJ, et al (2006) Least absolute regression network analysis of the murine osteoblast differentiation network. Bioinformatics 22: 477–484. 16332709 10.1093/bioinformatics/bti816

[17] Christley S, Nie Q, Xie X (2009) Incorporating Existing Network Information into Gene Network Inference. PLoS ONE 4: e6799. 19710931 10.1371/journal.pone.0006799 PMC2729382

[18] Xiong J, Zhou T (2012) Gene Regulatory Network Inference from Multifactorial Perturbation Data Using both Regression and Correlation Analyses. PLoS ONE 7: e43819. 23028471 10.1371/journal.pone.0043819 PMC3448649

[19] Gardner TS, Di Bernardo D, Lorenz D, Collins JJ (2003) Inferring genetic networks and identifying compound mode of action via expression profiling. Science 301: 102–105. 12843395 10.1126/science.1081900

[20] di Bernardo D, Thompson MJ, Gardner TS, Chobot SE, Eastwood EL, et al (2005) Chemogenomic profiling on a genome-wide scale using reverse-engineered gene networks. Nat Biotech 23: 377–383. 10.1038/nbt1075 15765094

[21] Wildenhain J, Crampin EJ (2006) Reconstructing gene regulatory networks: From random to scale-free connectivity. IEE Proceedings: Systems Biology 153: 247–256. 16986626 10.1049/ip-syb:20050092

[22] Rawool SB, Venkatesh KV (2007) Steady state approach to model gene regulatory networks—Simulation of microarray experiments. Biosystems 90: 636–655. 17382459 10.1016/j.biosystems.2007.02.003

[23] Srividhya J, Mourão MA, Crampin EJ, Schnell S (2009) Enzyme catalyzed reactions: From experiment to computational mechanism reconstruction. Computational Biology and Chemistry 34: 11–18. 19945917 10.1016/j.compbiolchem.2009.10.007

[24] Chang JT, Nevins JR (2006) GATHER: A systems approach to interpreting genomic signatures. Bioinformatics 22: 2926–2933. 17000751 10.1093/bioinformatics/btl483

[25] Bonneau R (2008) Learning biological networks: From modules to dynamics. Nature Chemical Biology 4: 658–664. 18936750 10.1038/nchembio.122

[26] Judd K, Mees A (1995) On selecting models for nonlinear time series. Physica D: Nonlinear Phenomena 82: 426–444.

[27] Crampin EJ, McSharry PE, Schnell S (2004) Extracting biochemical reaction kinetics from time series data. Lecture Notes in Computer Science (including subseries Lecture Notes in Artificial Intelligence and Lecture Notes in Bioinformatics). 329–336.

[28] Crampin EJ, Schnell S, McSharry PE (2004) Mathematical and computational techniques to deduce complex biochemical reaction mechanisms. Progress in Biophysics and Molecular Biology 86: 77–112. 15261526 10.1016/j.pbiomolbio.2004.04.002

[29] Mourão MA, Srividhya J, McSharry PE, Crampin EJ, Schnell S (2011) A Graphical User Interface for a Method to Infer Kinetics and Network Architecture (MIKANA). PLoS ONE 6: e27534. 22096591 10.1371/journal.pone.0027534 PMC3214083

[30] Alon U (2007) An Introduction to System Biology: Design Principles of Biological Circuits. New York: Chapman & Hall/CRC.

[31] Barabási A-L, Albert R (1999) Emergence of Scaling in Random Networks. Science 286: 509–512. 10521342 10.1126/science.286.5439.509

